# The Genuine Works of Hippocrates

**Published:** 1886-09

**Authors:** 


THE GENUINE WORKS OF HIPPOCRATES, translated from the Greek with a Preliminary Discourse and Annotations. By Francis Adams, LL. D., Surgeon. In two volumes. New York: Wm. Wood & Company. 1886.

These two volumes constitute the April and July numbers of Wood's Library of Standard Medical Authors.

The majority of medical men know of the writings of Hippocrates only by hearsay, which is equivalent to saying that they know them not at all. To such the perusal of these volumes will be a source of great pleasure and also of surprise. Hippocrates wrote, like Shakespeare, not for his own age, but for all ages. The truths which he gathered from his close observation and sagacious generalizations were based upon a study of natural phenomena untrammeled by any preconceived theories, and therefore are as true to-day as they were in the age of Pericles. Hence we have the marvelous phenomenon of a work which was written five hundred years before the time of Christ, and yet whose teachings seem less obsolete and ridiculous to-day than those of many authors of a century or two ago.

The commentaries and annotations which accompany the Hippocratic writings are well conceived and well written. They remove many of the obscurities which are inevitable in a work produced under a civilization so radically different from our own in all respects; yet they are so subordinated to the main theme as to present no appearance of offensive egotism, a not uncommon fault of such commentaries.

The publishers have earned the gratitude of the whole medical profession of America by placing within their reach this highly interesting work. V. O. H.

